# IGF2BP2 regulates DANCR by serving as an N6-methyladenosine reader

**DOI:** 10.1038/s41418-019-0461-z

**Published:** 2019-12-05

**Authors:** Xiaoge Hu, Wan-Xin Peng, Huaixiang Zhou, Jiahong Jiang, Xinchun Zhou, Dongsheng Huang, Yin-Yuan Mo, Liu Yang

**Affiliations:** 1 Key Laboratory of Tumor Molecular Diagnosis and Individualized Medicine of Zhejiang Province, Zhejiang Provincial People’s Hospital, People’s Hospital of Hangzhou Medical College, Hangzhou, 310014 Zhejiang China; 2 Cancer Institute, University of Mississippi Medical Center, Jackson, MS USA; 3 Department of Cell biology, School of Medicine, Jiangsu University, Zhenjiang, China; 4 Department of Pathology, University of Mississippi Medical Center, Jackson, MS USA; 5 Department of Pharmacology/Toxicology, University of Mississippi Medical Center, Jackson, MS USA

**Keywords:** Cancer, Molecular biology

## Abstract

The major function of Insulin-like growth factor 2 mRNA-binding protein 2 (IGF2BP2) is to regulate cell metabolism. However, emerging evidence indicates that IGF2BP2 plays a role in cancer, but the underlying mechanism is largely unknown. Here we showed that upregulation of IGF2BP2 is associated with poor outcomes of pancreatic cancer patients and suppression of IGF2BP2 inhibits cell proliferation. We further showed that IGF2BP2 regulates lncRNA DANCR. Ectopic expression IGF2BP2 enhances, whereas knockdown (KD) or knockout (KO) of IGF2BP2 suppresses DANCR expression. Moreover, in vivo RNA precipitation and reciprocal RNA immunoprecipitation revealed that IGF2BP2 interacts with DANCR. DANCR promotes cell proliferation and stemness-like properties. Experiments with xenograft models revealed that while ectopic expression of DANCR promotes, DANCR KO suppresses tumor growth. Mechanistically, DANCR is modified at N6-methyladenosine (m6A) and mutagenesis assay identified that adenosine at 664 of DANCR is critical to the interaction between IGF2BP2 and DANCR where IGF2BP2 serves a reader for m6A modified DANCR and stabilizes DANCR RNA. Together, these results suggest that DANCR is a novel target for IGF2BP2 through m6A modification, and IGF2BP2 and DANCR work together to promote cancer stemness-like properties and pancreatic cancer pathogenesis.

## Introduction

Insulin-like growth factor 2 (IGF2) mRNA-binding protein 2 (IGF2BP2) is an RNA-binding protein (RBP) and serves as a posttranscriptional regulatory factor for mRNA localization, stability, and translational control. Dysregulation of IGF2BP2 is often associated with human diseases such as insulin resistance, diabetes, or cancer [1]. Our previous study showed that IGF2BP2 regulates colorectal cancer cell proliferation through suppressing the miR-195-mediated RAF-1 degradation [2]. Of interest, IGF2BP2 has been implicated in maintaining glioblastoma stem cell (GSC) properties through regulating let-7-mediated gene silencing [3]. Experiments with IGF2BP2 knockout (KO) mice demonstrated that IGF2BP2 is a tumor promoter that drives cancer progression [4]. Recently, IGF2BP family proteins, including IGF2BP2, have been shown to be required for their recognition of N6-methyladenosine (m6A) RNA modifications and are critical for their oncogenic functions [5].

It is well known that RNA is subject to a variety of modifications, among which internal modifications play important roles in RNA metabolism. The most abundant internal RNA modification is N6-methyladenosine (m6A) which can tag thousands of mRNAs in mammalian cells. The m6A modification machinery consists of “writers”, “readers” and “erasers” [6] and they are delicately balanced to support normal cellular functions. In this regard, readers are those that can recognize m6A and bring the methylated RNAs with various functional consequences, including translation, mRNA stability, alternative splicing, RNA processing, nuclear trafficking, microRNA binding and RNA-protein interaction [7]. Two large families of RNA-binding proteins (RBPs) can serve as readers and they are the YTH domain family (YTHDF) [8] and IGF2BPs [5]. In contrast to YTHDF that may destabilize the recognized RNA, IGF2BPs serve as a distinct family of m6A readers that target large numbers of mRNA transcripts, and promote the stability and storage of their target mRNAs in an m6A-dependent manner under normal and stress conditions. However, it remains to be determined as to whether IGF2BP2 can regulate expression of long-non-coding RNAs (lncRNAs) through m6A modifications, leading to self-renewal of cancer stem cells (CSCs) in pancreatic cancer.

In the present study, we found that IGF2BP2 is highly expressed in pancreatic cancer, and upregulation of IGF2BP2 is associated with poor prognosis of pancreatic cancer patients. We showed that lncRNA DANCR is a target of IGF2BP2. Further characterization revealed that DANCR promotes the cell proliferation, stemness-like properties as well as tumorigenesis of pancreatic cancer cells. Importantly, we found that IGF2BP2 regulates DANCR stability by serving as a reader for m6A-modified DANCR.

## Results

### IGF2BP2 is a potential unfavorable prognostic marker in pancreatic cancer

**Fig. 1:**
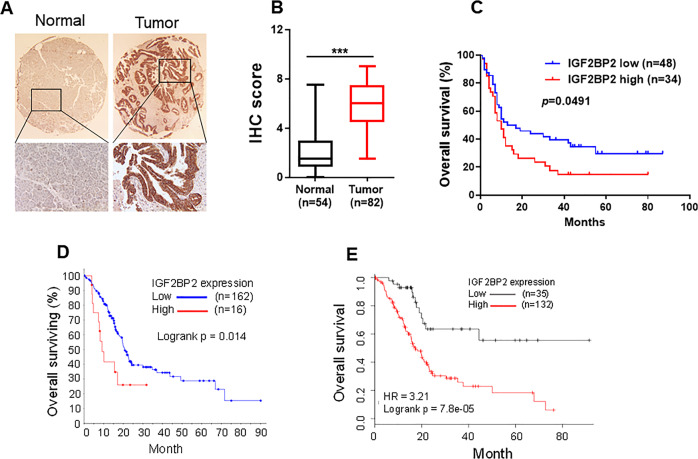
IGF2BP2 is highly expressed in pancreatic cancer, and is associated with clinical outcomes. **a** Representative immunohistochemistry (IHC) results of IGF2BP2 protein expression from the pancreatic cancer tissue microarray. **b** Statistical analysis of IHC expression in normal and tumor specimens. **c** Kaplan–Meier plotter for overall survival based on IGF2BP2 protein expression. **d** Overall survival based on Pancreatic Adenocarcinoma dataset (TCGA, Provisional) analysis of mRNA levels (high and low). **e** Analysis of Pan-Cancer RNA seq (pancreatic adenocarcinoma dataset) from Kaplan–Meier plotter (http://kmplot.com/analysis/) also suggests poor overall survival with high expression of IGF2BP2 as compared with the low IGF2BP2 expression group.

To investigate the clinical importance of IGF2BP2 in pancreatic cancer, we first determined IGF2BP2 expression by immunohistochemistry (IHC) staining in the pancreatic cancer tissue microarray (TMA) slide containing 82 pancreatic cancer and 54 normal samples (Supplementary Table 2). As compared to normal tissue, IGF2BP2 was significantly upregulated in tumor tissue at the protein level (Figs. 1a, b; S1A) and this upregulation of IGF2BP2 was an unfavorable marker for cancer patients (Fig. 1c). Clinical outcomes related to disease grade, stage and age were shown in Fig. S1B–D. Moreover, interrogation of TCGA pancreatic adenocarcinoma RNA-seq dataset through cBioPortal (http://www.cbioportal.org/) [9] revealed that a high level of IGF2BP2 was significantly associated with poor overall survival (OS) (Fig. 1d) and disease free survival (Fig. S2). Consistent with this, analysis of Pan-Cancer RNA seq (pancreatic adenocarcinoma dataset) from Kaplan–Meier plotter (www.KMplot.com) also supported that IGF2BP2 was a potential unfavorable prognostic marker for pancreatic adenocarcinoma (Fig. 1e). These results highlight the clinical significance of IGF2BP2 in pancreatic cancer.

### IGF2BP2 promotes pancreatic cancer cell proliferation

**Fig. 2:**
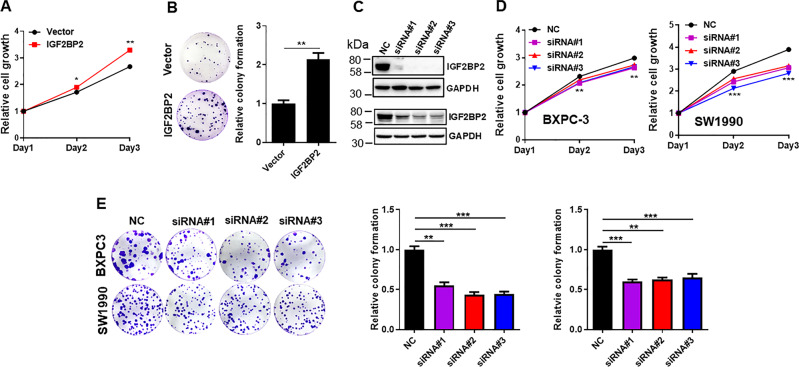
IGF2BP2 promotes pancreatic cancer cell proliferation. **a** Ectopic expression of IGF2BP2 promotes cell proliferation in BXPC-3 cells as detected by MTT assays. **b** IGF2BP2 promote colony formation in BXPC-3 cells. **c** Suppression of IGF2BP2 by IGF2BP2-siRNAs (siRNA#1, siRNA#2, siRNA#3) as determined by western blot. Top, BXPC-3; bottom, SW1990. **d** In contrast to ectopic expression, IGF2BP2 siRNAs significantly suppresses cell growth in BXPC-3 and SW1990 cells. **e** IGF2BP2 silencing significantly reduces colony formation in BXPC-3 and SW1990 cells. Values in **a**, **b**, **d**, **e** are mean ± SEM. **P < 0.01; ***P < 0.001.

To determine the role of IGF2BP2 in pancreatic cancer, we ectopically expressed IGF2BP2 in BXPC-3 cells (Fig. S3A) and found that IGF2BP2 promoted cell proliferation, as detected by MTT assays (Fig. 2a) or cell survival as determined by colony formation assays (Fig. 2b). By contrast, knockdown by IGF2BP2 specific siRNAs (Fig. 2c) reduced the proliferation of BXPC-3 cells (Fig. 2d, left). A similar effect was also seen in SW1990 cells (Fig. 2d, right). IGF2BP2 siRNAs also suppressed colony formation in both cell lines (Fig. 2e).

**Fig. 3:**
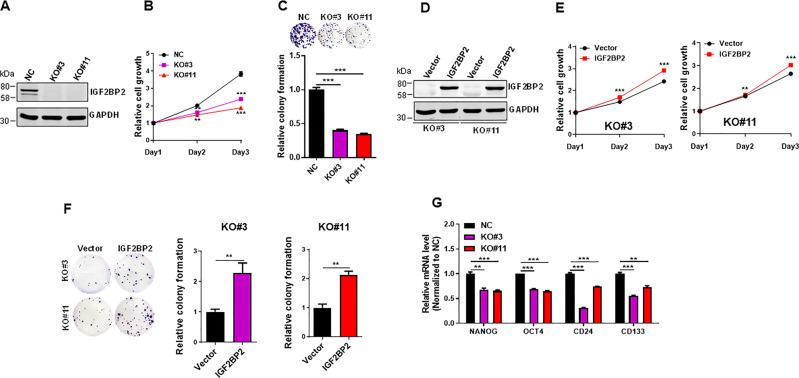
IGF2BP2 KO suppresses pancreatic cancer proliferation, which can be rescued by re-expression of IGF2BP2 in the KO cells. **a** KO of IGF2BP2 in BXPC-3 cells as verified by western blot. **b** IGF2BP2 KO suppresses cell proliferation in BXPC-3 cells as determined by MTT assay. **c** IGF2BP2 KO suppresses colony formation in BXPC-3 cells. **d** Re-expression of IGF2BP2 in IGF2BP2 KO cells as determined by western blot. **e** Rescue of IGF2BP2 promotes cell proliferation in two IGF2BP2 KO cell lines. **f** Rescue of IGF2BP2 increases colony formation in two IGF2BP2 KO cell lines. **g** IGF2BP2 KO suppresses stemness-related genes (OCT4, NANOG, CD24, and CD133), as determined by qRT-PCR. Values in **b**, **c**, **e**, **f**, **g** are mean ± SEM. **P < 0.01; ***P < 0.001.

To better characterize IGF2BP2 function in pancreatic cancer cells, we generated IGF2BP2 KO in BXPC-3 cells and selected two KO clones (KO#3 and KO#11) for characterization (Fig. 3a). IGF2BP2 KO caused a significant reduction of cell proliferation, as detected by MTT assay (Fig. 3b) or colony formation (Fig. 3c). To further demonstrate the role of IGF2BP2 in proliferation of pancreatic cancer cells, we performed a rescue, i.e., re-expression of IGF2BP2 in the KO cells (Fig. 3d). As expected, re-expression of IGF2BP2 restored the cell proliferation ability (Fig. 3e) and colony formation ability (Fig. 3f). Finally, IGF2BP2 KO caused downregulation of stemness-related genes such as OCT4 and NANOG (Fig. 3g).

### IGF2BP2 regulates DANCR expression

**Fig. 4:**
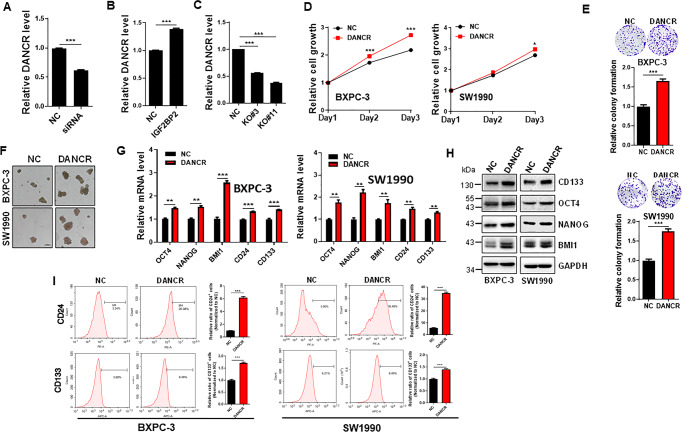
IGF2BP2-mediated upregulation of DANCR promotes cell proliferation and stemness-like properties. **a** IGF2BP2 siRNA down regulates DANCR expression in BXPC-3 cells, as determined by qRT-PCR, whereas ectopic expression of IGF2BP2 increases DANCR level (**b**). **c** Like IGF2BP2 siRNA, IGF2BP2 KO also suppresses DANCR expression in BXPC-3 cells. **d** DANCR promotes cell proliferation in BXPC-3 and SW1990 cells, as determined by CCK8 assays. **e** DANCR also promotes colony formation. **f** Ectopic expression of DANCR increases sphere formation in number and size. **g** DANCR-overexpression promotes expression of stemness-related genes (OCT4, NANOG, BMI 1, CD24, and CD133) in pancreatic cancer cells, as determined by qRT-PCR and at the protein level (**h**). **i** Percentage of CD24^+^ and CD133^+^ population in DANCR overexpression and control (NC) cells, as determined by flow cytometry. The statistical results of flow cytometry were showed on the right. Values in **a**, **b**, **c**, **d**, **e**, **g**, **i** are mean ± SEM. *P < 0.05; **P < 0.01; ***P < 0.001.

Next, we determined whether IGF2BP2 can regulate expression of lncRNAs because lncRNAs have been shown to function as master gene regulators and play an important role in cancer initiation, progression, and metastasis as well as stem cell maintenance [10–15]. Thus, we performed profiling using RT-PCR lncRNA profiler [16] in IGF2BP2 knockdown cells and found that several lncRNAs were either downregulated or upregulated as compared to control. Among them was DANCR, which was downregulated about 40% by IGF2BP2 knockdown (Fig. 4a). In contrast, IGF2BP2 overexpression upregulated DANCR (Fig. 4b). Furthermore, IGF2BP2 KO suppressed DANCR expression (Fig. 4c). Since DANCR has also been reported to promote cancer stemness-like properties [17, 18], we focused on DANCR in this study.

### DANCR promotes cell proliferation and stemness-like properties

To determine whether DANCR is involved in the IGF2PB2-mediated pancreatic cancer progression and stemness-like properties, we ectopically expressed DANCR in BXPC-3 and SW1990 cells (Fig. S3B). The CCK8 assays revealed that overexpression of DANCR promoted cell proliferation (Fig. 4d). Furthermore, DANCR also enhanced colony formation (Fig. 4e).

We then determined the effect of DANCR on sphere formation because enhanced sphere formation is an important property for CSCs [19, 20]. Ectopic expression of DANCR in BXPC-3 and SW1990 cells increased the sphere formation; spheres derived from DANCR overexpression were not only larger in size, but also the number of spheres was more than vector control cells (Fig. 4f). Consistent with this result, the stemness-related genes (OCT4, NANOG, BMI1, CD24, and CD133) were significantly upregulated in DANCR overexpression at the mRNA level as detected by qRT-PCR (Fig. 4g) or protein level as detected by western blot (Figs. 4h and S4A). CD24 and CD133 are known surface markers for pancreatic CSCs [21, 22]. FACS analysis detected a substantial upregulation of CD24^+^ and CD133^+^ cell population when DANCR was overexpressed (Fig. 4i).

**Fig. 5:**
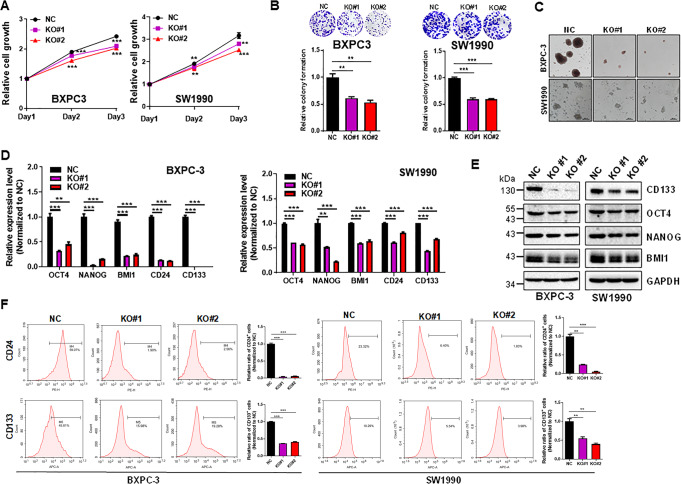
DANCR KO suppresses cell proliferation and stemness-like properties of pancreatic cancer cells. **a** DANCR KO suppresses cell proliferation in BXPC-3 and SW1990 cells, as determined by CCK8 assays. **b** DANCR KO suppresses colony formation. **c** DANCR KO inhibits sphere formation in number and size. **d** DANCR KO suppresses stemness-related genes in pancreatic cancer cells, as determined by qRT-PCR and at the protein level **e**. **f** Percentage of CD24^+^ and CD133^+^ population in DANCR KO and control (NC) cells, as determined by flow cytometry. The statistical results of flow cytometry were showed on the right. Values in **a**, **b**, **d**, **e**, **f** are mean ± SEM. *P < 0.05; **P < 0.01; ***P < 0.001.

To further confirm the role of DANCR in promoting stemness-like properties, we generated DANCR KO in BXPC-3 and SW1990 cells through CRISPR/Cpf1 system via dual gRNA approach [23] (Fig. S3C). In contrast to overexpression, DANCR KO suppressed cell proliferation (Fig. 5a) and colony formation (Fig. 5b) in both cell lines. Furthermore, DANCR KO suppressed the number of tumor spheres and these spheres were much smaller than vector control (Fig. 5c). In addition, stemness-related genes (OCT4, NANOG, BMI1, CD24, and CD133) were significantly downregulated at the mRNA (Fig. 5d) or protein levels (Fig. 5e and Fig. S4B). Finally, the populations of CD24^+^ cells and CD133^+^ cells were also decreased after DANCR KO in these two cell lines (Fig. 5f). Taken together, these results suggest that DANCR promotes stemness-like properties of pancreatic cancer cells.

### DANCR promotes tumorigenesis of pancreatic cancer cells

**Fig. 6:**
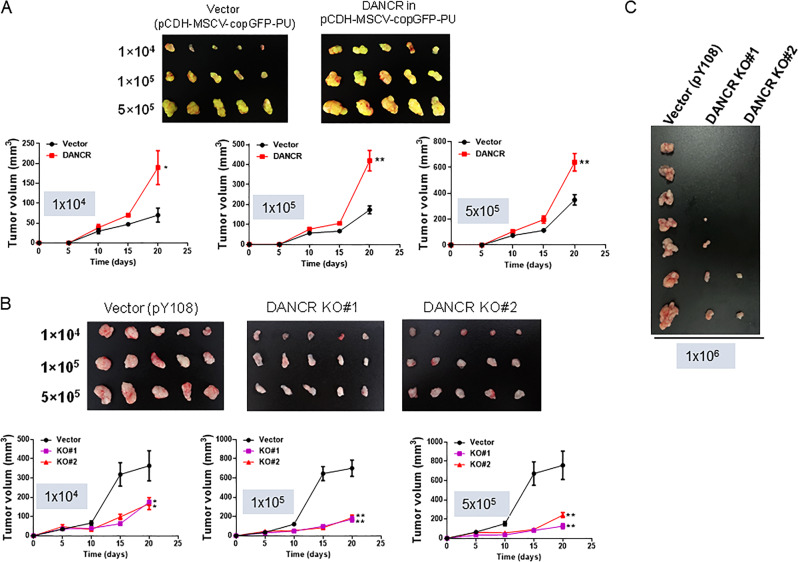
DANCR overexpression promotes, whereas DANCR KO suppresses tumor progression. **a** DANCR promotes the tumor growth of BXPC-3 cells. DANCR overexpression (DANCR) and vector control (pCDH-MSCV-copGFP-T2A-Pu) cells were subcutaneously injected into the nude mice with 1 × 10^4^, 1 × 10^5^, 5 × 10^5^ cells per mouse as indicated in “Materials and methods”. **b** DANCR KO significantly suppresses the tumor growth rate of BXPC-3 cells. DANCR KO and vector control (pY108) cells were subcutaneously injected into the nude mice with 1 × 10^4^, 1 × 10^5^, 5 × 10^5^ cells per mouse as in (**a**). (**c**) DANCR KO inhibits tumorigenesis of BXPC-3 cells in orthotopic pancreatic tumor mouse model. DANCR KO and vector control cells were injected into the pancreas of nude mice with 1 × 10^6^ cells per mouse as detailed in “Materials and methods”. Values in **a**, **b** are mean ± SEM. *P* < 0.05; **P < 0.01.

To determine the effect of DANCR on pancreatic cancer tumorigenesis, we tested three different numbers of cancer cells (1 × 10^4^ cells/mouse; 1 × 10^5^ cells/mouse and 5 × 10^5^ cells/mouse). DANCR overexpression in BXPC-3 cells significantly increased tumor growth rate and tumor size as compared to vector control (Figs. 6a and S5A). By contrast, DANCR KO suppressed tumor growth and tumor size for three different numbers of cells (Figs. 6b and S5B). Furthermore, we performed an orthotopic model by injecting 1 × 10^6^ cells into pancreas directly. The suppression of tumor growth by DANCR KO in this orthotopic model was even greater than in subcutaneous injection (Fig. 6c vs 6b). These results further suggest the importance of DANCR in pancreatic cancer progression.

### IGF2BP2 interacts with DANCR and regulates its stability

**Fig. 7:**
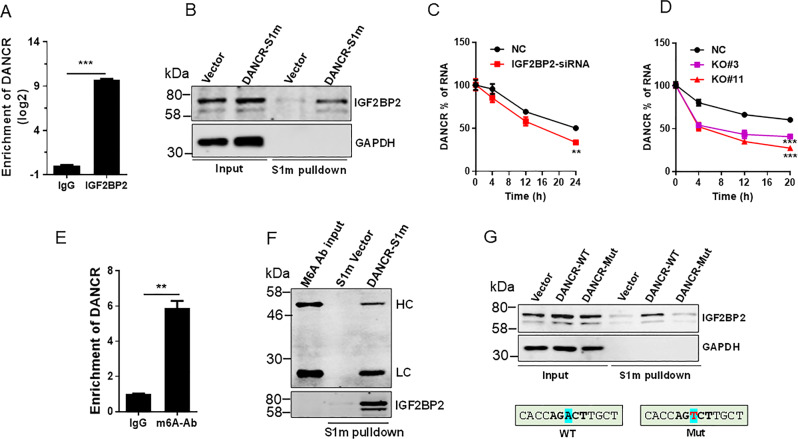
IGF2BP2 regulates DANCR mRNA stability through m6A modification at A664. **a** IGF2BP2 interacts with DANCR, as determined by RIP assay using IGF2BP2 antibody. **b** Detection of in vivo interaction between IGF2BP2 and DANCR by S1m tagged DANCR pulldown. In vivo expressed S1m tagged DANCR was first pulled down by streptavidin beads and IGF2BP2 was then detected by western blot. **c** IGF2BP2 siRNA reduces DANCR stability in BXPC-3 cells. Cells were first transfected with IGF2BP2 siRNA#3 and control siRNA. Then, the transfected cells were treated with 2 µg/ml actinomycin D and total RNA was isolated at various time points as indicated. **d** IGF2BP2 KO also reduces DANCR stability. **e** Enrichment of DANCR by RIP assay with m6A antibody. **f** Detection of interaction of DANCR with m6A antibody. In vivo expressed S1m tagged DANCR was first pulled down by streptavidin beads and then incubated with m6A antibody. The signals bound to the membrane were detected by donkey anti-mouse antibody conjugated with IRDye^®^ 680RD. HL heavy chain, LC light chain. **g** Mutation A664 in DANCR abolishes its interaction with m6A antibody. WT wild type, Mut mutant. Values in **a**, **c**, **d**, **e** are mean ± SEM. **P < 0.01; ***P < 0.001.

To determine the underlying mechanism of IGF2PB2-mediated DANCR expression, we asked whether IGF2PB2 interacts with DANCR because as a RBP, IGF2PB2 can interact with large numbers of RNAs. RNA immunoprecipitation (RIP) assays with IGF2PB2 antibody showed over a 1000-fold enrichment of DANCR as compared to IgG control (Fig. 7a). To further verify their interaction, we performed in vivo RNA precipitation assay. In this case, we tagged DANCR with a modified S1 (S1m) [24] which was derived from a streptavidin-binding aptamer termed S1 [25, 26] that acts like biotin. This S1m greatly improves its affinity for streptavidin [24]. S1m tagged DANCR was expressed in the cell and then was pulled down by streptavidin beads from the cellular extract (Fig. S6A). The advantage of this tagging approach over the in vitro biotin labeling approach is that we are able to detect protein-RNA interaction in the native context. Thus, we transfected BXPC-3 cells with S1m tagged DANCR and the cellular extract was used for pulldown assays. Western blot detected a specific band of IGF2BP2 (Fig. 7b). To determine how IGF2BP2 regulates DANCR expression, we examined the DANCR stability because IGF2BP2 has been implicated in regulating RNA stability [5]. BXPC-3 cells were transfected with IGF2BP2-siRNA or negative control siRNA and then treated with actinomycin D at 2 µg/ml for various time points before total RNA was extracted. As shown in Fig. 7c, DANCR decay in the IGF2BP2-siRNA treated cells was faster than control siRNA-treated cells. Furthermore, IGF2BP2 KO also caused a faster decay of DANCR RNA than vector control (Fig. 7d), suggesting that IGF2BP2 can increase DANCR RNA stability. We also determined the effect of ectopic expression of DANCR on cell proliferation in IGF2BP2 KO cells and found that ectopic expression of DANCR enhanced cell proliferation and viability in the IGF2BP2 KO cells (Fig. S7), suggesting that IGF2BP2 promotes cell proliferation and survival in part through DANCR.

IGF2BP family, including IGF2BP2, has been recently shown to be involved in RNA methylation pathway and function as RNA stabilizers [5]. To determine whether DANCR is subject to RNA methylation (m6A) such that the methylated DANCR is recognized by IGF2BP2, we performed RIP assays with m6A antibody and identified ~a sixfold enrichment of DANCR over the IgG control (Fig. 7e). To further show the m6A modification of DANCR, we performed a special reciprocal RNA precipitation (Fig. S6B). In this case, we first transfected BXPC-3 cells with S1m-tagged DANCR or vector control, and then recovered S1m-tagged DANCR RNA by pulldown with streptavidin beads. Next, we added m6A antibody to the recovered RNA solution containing the beads and incubated overnight at 4 °C, followed by five times of washes. Finally, the precipitated pellets were resuspended in protein sample buffer, followed by western blot with second antibody against mouse IgG (H + L). As expected, we detected both heavy chain and light chain in the DANCR-S1m lane, but not the vector control lane (Fig. 7f), further supporting that DANCR is methylated at m6A. Examining the DANCR sequences, we found a potential m6A motif [27] at nt 664. Thus, we changed this “A” to “T” (Fig. 7g, bottom). S1m precipitation detected the IGF2BP2 band in the WT lane, but this band was substantially decreased in the mutant lane (Fig. 7g). Together, these results suggest that like many other mRNAs, DANCR can also be methylated at m6A; IGF2BP2 serves as a reader for the methylated DANCR to increase its stability.

## Discussion

IGF2BP2 is implicated in regulation of mRNA localization and mRNA stability, impacting cell proliferation, invasion, epithelial-mesenchymal transition and CSC maintenance. As a RBP, IGF2BP2 interacts with large numbers of RNAs. In particular, IGF2BP2 can serve as a reader for m6A, a predominant RNA modification which has been shown to have a fundamental effect on various cellular processes. Evidence indicates that most of these m6A modified RNAs are mRNAs whereas m6A modified lncRNAs account for a very small portion [27, 28]. Here, we provide evidence that DANCR is a novel m6A modified target that can be recognized by IGF2BP2, leading to an increased stability of DANCR in pancreatic cancer.

Our study supports the clinical significance of IGF2BP2 in pancreatic cancer. We first showed upregulation of IGF2BP2 in tumor specimens as compared to normal tissue by IHC analysis of pancreatic cancer TMA. Interrogation of TCGA pancreatic cancer RNA seq dataset revealed upregulation of IGF2BP2. Importantly, this upregulation of IGF2BP2 is significantly associated with poor patient outcomes. This is also supported by analysis of Pan-Cancer RNA seq (pancreatic adenocarcinoma dataset) from Kaplan Meier plotter. By overexpression, knockdown, KO and rescue approaches, we showed that IGF2BP2 promotes cell proliferation and viability, and stemness-like properties.

DANCR was first identified as an antidifferentiation lncRNA and loss of DANCR in progenitor cells causes rapid induction of differentiation genes [29]. Later, DANCR was found to be important in supporting cancer cell stemness in hepatocellular carcinoma (HCC) and osteosarcoma [17, 18]. For example, DANCR is able to upregulate CTNNB1 in HCC [17], and activate AKT pathway in osteosarcoma [18]. Consistent with these findings, DANCR is upregulated in a variety of cancer types including nasopharyngeal carcinoma, bladder cancer, breast cancer, prostate and ovarian cancer [30–33]. These studies suggest that a major mechanism for DANCR to regulate gene expression is to function as a microRNA sponge, i.e., competitive endogenous RNA mechanism [34]. In addition, DANCR can increase HIF-1α mRNA stability through interacting with the NF90/NF45 complex [30]; it activates IL-11-STAT3 signaling and increases cyclin D1 and PLAU expression via guiding leucine-rich pentatricopeptide repeat containing (LRPPRC) to stabilize mRNA [31]. In breast cancer, DANCR upregulates PI3K/AKT signaling through activating serine phosphorylation of RXRA [33]. However, little is known about the function of DANCR in pancreatic cancer, and in particular, it is not known whether DANCR is subject to m6A modification.

Role of m6A modifications in cancer has been demonstrated in various types of cancer such as leukemia, brain cancer, breast cancer, and lung cancer. For example, the m6A eraser FTO is highly expressed in acute myeloid leukemia (AML). Forced expression of FTO significantly enhances the viability and growth of human AML cells through demethylation of ASB2 and RARA, two critical targets genes of FTO. Knockdown of FTO causes an increase in the m6A levels on ASB2 and RARA [35]. ALKBH5 is another important eraser in RNA methylation machinery. Upregulation of ALKBH5 is associated with poor prognosis for the patients with glioblastoma. ALKBH5 knockdown increases m6A modification on its target FOXM1, a critical factor for self-renewal and tumorigenesis of GSCs. Therefore, ALKBH5-mediated upregulation of FOXM1 expression enhances GSC self-renewal and proliferation [36]. In addition, ALKBH5 promotes expression of NANOG, a well-known factor for the self-renewal process of undifferentiated ESCs, and thus it plays a role in primary tumor formation and metastasis [37]. Consistent with this, overexpression of m6A writer METTL3 or inhibition of FTO has been shown to suppress GSC growth and self-renewal [38]. Apparently, these studies suggest that RNA methylation impacts cancer cell stemness and progression through the interplay among writers, erasers and readers. The present study suggests that m6A-mediated upregulation of DANCR promotes stemness like properties in pancreatic cancer.

We took advantage of S1m in vivo pulldown, demonstrating that DANCR is subject to m6A modification. We showed that m6A modification increases the stability of DANCR and A664 is critical for the interaction between DANCR and IGF2BP2. In the literature, RNA precipitation assays often use in vitro synthesized RNA probes to pulldown protein binding partners. In these assays, the probes are added to cellular extract. In other words, the pulled down proteins are mostly like those in free form whereas the bound protein is less likely to be pulled down, especially for those RNAs that are post-transcriptionally modified. Therefore, such assays may not recapitulate RNA-protein interactions in the cell. In contrast, the in vivo tagging approach presented in this study enables us to better reveal their true interactions because the probe RNA is expressed in the cell. Furthermore, synthesis of long RNA probes carrying a modification such as m6A is almost impossible whereas S1m tagged RNA can be modified in the cell just like the endogenous counterpart. Importantly, with this approach we are able to test the function of mutant DANCR vs wild type DANCR (Fig. 7f), as we showed that mutating A664 of DANCR abolishes its interaction with IGF2BP2.

In summary, we found that upregulation of IGF2BP2 in pancreatic cancer is associated with poor clinical outcomes. Our study further suggests that IGF2BP2 functions in partnerships with DANCR to regulate its stability. In normal cells, the level of IGF2BP2 is lower, and thus, its ability to interact with and stabilize DANCR is limited. In tumor cells, IGF2BP2 is upregulated, which increases the chance of IGF2PB2 to interact with and stabilize DANCR, especially, when RNA methylation machinery is dysregulated. As a result, these tumor cells become more proliferative and more resistant to anticancer therapy. Therefore, IGF2BP2-DANCR axis may not only serve as a valuable biomarker, but also provide opportunities for therapeutic intervention in pancreatic cancer.

## Materials and methods

### Reagents

CD24-PE (Cat #12-0247-42) and CD133-APC (Cat.#17-1338-42) antibodies were obtained from ThermoFisher Scientific (Waltham, MA). Antibodies against IGF2BP2 (Cat #14672), CD133 (Cat #86781), OCT4 (Cat #2750), NANOG (Cat #4903), and BMI1 (Cat #6964) for western blot were purchased from Cell Signaling (Danvers, MA). Antibody against IGF2BP2 (Cat #11601-AP) for IHC was bought from ProteinTech (Rosemont, IL). The siRNA sequence targeting IGF2BP2 were designed and synthesized by Genepharma Company (Shanghai, China). Primers were purchased from IDT (Coralville, IA). The following constructs were obtained from Addgene (Watertown, MA): pY108 (lenti-AsCpf1, #84739); LentiCRISPR v2 (#52961); MSCV-human Igf2bp2-IRES-GFG (#91890). pCDH-MSCV-copGFP-T2A-Pu was purchased from System Biosciences (Mountain View, CA).

### Cell culture

Human pancreatic cancer cell line BXPC-3 and SW1990 were purchased from American Type Culture Collection (Manassas, VA, USA), and they were grown in RPMI-1640 medium (ThermoFisher Scientific Pittsburgh, PA, USA) and DMEM medium (ThermoFisher Scientific), respectively, supplemented with 10% fetal bovine serum (ThermoFisher Scientific). BXPC-3 and SW1990 were authenticated by short tandem repeat analysis (Shanghai Biowing Applied Biotechnology, Shanghai) and were regularly tested for Mycoplasma contamination by PCR.

### Transfection

Cells were transfected with siRNAs or control siRNAs using Lipofectamine 3000 transfection reagent (Life Technologies) according to the manufacturer’s instruction. The siRNA sequences targeting IGF2BP2 were designed and synthesized by Genepharma Company (Shanghai, China), and they were listed in Supplementary Table 1.

### Quantitative RT-PCR

Total RNA was extracted from cells using Trizol reagent (Invitrogen) according to the manufacturer’s instruction. cDNA was subsequently synthesized using PrimeScript^TM^ RT Master Mix kit (Takara). Real time PCR reaction was performed using SYBR Premix Ex Taq^TM^, (Tli RNaseH Plus) kit (Takara). The primers were listed in Supplementary Table 1.

### Cell proliferation assay

Cell proliferation was analyzed using MTT assays as described previously [39] or Cell Counting Kit-8 (CCK-8, Biotool) according to the manufacturer’s instruction. Briefly, BXPC-3 or SW1990 cells were seeded in 96-well plates at a density of 3.5 × 10^3^ cells per well. The old medium was removed, and then 100 µl fresh medium containing 1/10 volume of CCK-8 was added to wells at 24, 48, 72 h, respectively. Absorbance at 450 nm was measured after incubation at 37 °C for 4 h.

### Colony formation assay

For the colony formation assay, cells were seeded in six-well plates. After 8–14 days, the cells were fixed with 4% PFA (Sigma) and then stained with crystal violet. The number of colonies was countered for five representative fields. The experiments were repeated for three times. Relative colony formation was calculated as compared with vector controls as 100%.

### Western blot

Cells were harvested, and proteins were extracted and quantified as previously described [40]. Protein samples were separated in a polyacrylamide SDS gel before transferring to PVDF membrane. After probing with a primary antibody, the membrane was incubated with a secondary antibody labeled with either IRDye 800CW or IRDye 680. Finally, signal intensity was determined using the Odyssey Infrared Imaging System (LI-COR Biosciences, Lincoln, NE, USA). Alternatively, we also used the chemiluminescence detection system, ChemiDoc^TM^ MP Imaging System (Bio-Rad) to determine the relative protein levels.

### Plasmid construction

DANCR for ectopic expression experiments was cloned into pCDH-MSCV-copGFP-T2A-Pu. We first amplified DANCR by PCR using primers DANCR-RI-5.1 and DANCR-NotI-3.1, and then cloned into pCDH-MSCV-copGFP-T2A-Pu at EcoR I and Not I sites using NEBuilder^®^ HiFi DNA Assembly kit (New England Biolabs, Ipswich, MA). S1m expression vector carried four copies of modified S1 sequences (Supplementary Table 1); these four copies were sequentially cloned into pCDH-CAG-EF1-copGFP-T2A-Pu (derived from pCDH-MSCV-copGFP-T2A-Pu) at EcoR1 and BamH1 sites, resulting in S1m tagging vector, pCDH-CAG-4xS1m-EF1-copGFP-T2A-Pu (Supplementary materials). DANCR was first amplified using primers DANCR-S1m-R1-5.1 and DANCR-S1m-BamH1-3.1 and then cloned into this S1m expression vector at EcoR I and BamH I sites. Mutation of DANCR at a putative m6A site was made by two overlapped PCR products using primers DANCR-S1m-R1-5.1 and DANCR-mut-3.1; DANCR-mut-5.1 and DANCR-S1m-BamH1-3.1 (Supplementary Table 1), and then cloned into S1m vector at EcoR I and BamH I sites. PCR reactions for cloning purpose used Phusion enzyme (ThermoFisher Scientific). All PCR products were verified by DNA sequencing. (4 × S1m expression vector and DANCR WT/Mutant in S1m expression vector will be available from Addgene.)

### KO of IGF2BP2 and DANCR

IGF2BP2 was knocked out using CRISPR/Cas9 dual gRNA approach as described previously [23]. Two gRNAs targeting the first exon of IGF2BP2 were designed through Benchling (https://benchling.com). For cloning dual gRNAs, we modified LentiCRISPR v2 [41] by using the optimized scaffold [42] to generate LCV2-m. Two gRNAs and mouse U6 were introduced by PCR using primers IGF2BP2-T1a-5.1 and IGF2BP2-T1b-3.1, and mouse U6 as a template. Finally this PCR product was cloned into LCV2-m at BsmB1 site using NEBuilder^®^ HiFi DNA Assembly kit. For DANCR KO, we used CRISPR/Cpf1 dual gRNA approach. Dual gRNAs targeting outside regions of DANCR was designed through Benchling (https://www.benchling.com/). Oligonucleotides for dual gRNAs were cloned into pY108 at BsmB1 site [43]. All primer sequences were listed in Supplementary Table 1.

We introduced empty vector or dual gRNA expression vector into BXPC-3 cells by infection. Three days later, the infected cells were subject to puromycin (1 μg/ml) selection for 10 days. Individual puromycin resistant colonies were picked up manually and then expanded in 12-well plates. Initial identification of KO clones was carried out by genomic PCR. The potential clones were further verified by qRT-PCR.

### Immunohistochemistry

Pancreatic cancer TMA slide containing 82 pancreatic cancer and 54 normal samples was purchased from OUTDO BIOTECH (Shanghai, China). The slide was deparaffinized, rehydrated, and antigens were retrieved by Antigen Retrieval Citra Plus Solution (BioGenex, #HK080-9K), followed by treatment with 3% H_2_O_2_ to block endogenous peroxidases. After primary IGF2BP2 antibody, and secondary antibody, and ABC staining using VECTASTAIN^®^ Elite^®^ ABC HRP Kit (VectaStain, #PK-6101), the signals were revealed by ImmPACT^TM^ DAB (Vector Laboratories, #SK-4105). The overall IGF2BP2 expression was calculated from total score (signaling intensity score x staining distribution score). The signal intensity scores were: 0 (no signal), 1 (weak), 2 (moderate), and 3 (strong). The staining distribution scores were based on the percentage of positive cells: 0 (0%), 1 (1–10%), 2 (10–50%), and 3 (51–100%). Clinical and histopathological information of patients were shown in Supplementary Table 2.

### Sphere formation assay

Cells were seeded at a density of 3 × 10^4^ cells and cultured in serum-free DME/F-12 (1:1) medium (ThermoFisher) supplemented with 10 ng/ml epidermal growth factor,10 ng/ml basic fibroblast growth factor and N2 (ThermoFisher) on ultralow attachment six-well plates (Corning, Corning, NY). Medium was replaced every three days. The spheres were cultured for 2 weeks, and then pictured and counted.

### Flow cytometric analysis

A total of 1 × 10^6^ cells were incubated with CD24 or CD133 antibody (1:1000) in 1× PBS for 30 min at 4 °C and then washed with 1× PBS for three times. The cells were then analyzed by flow cytometry (ACEA NovoCyte^TM^).

### RNA immunoprecipitation (RIP)

We used IGF2BP2 antibody to pull down DANCR. The IGF2BP2 antibody was then recovered with protein A/G beads. The RNA level of DANCR in the precipitates was measured by qRT-PCR. For m6A RIP, we used m6A antibody (MABE1006) (MilliporeSigma, Burlington, MA) to pull down m6A modified DANCR. Total RNA was isolated from a 10 cm dish culture and dissolved in 50 µl RNase-free water, of which 5 µl was saved as RNA input the remaining 45 µl RNA was added to 500 µl pulldown lysis buffer containing RNase inhibitor. The total RNA were first incubated briefly with 1 µl mouse IgG and then the IgG was removed by protein A/G beads. The pre-cleaned lysates were transferred to new tubes and mixed with mouse IgG or m6A antibody. The tubes were then rotated overnight at 4 °C, followed by incubation with protein A/G beads under the same condition overnight. Finally, m6A bound RNA was extracted with Trizol and the RNA level of DANCR was measured by qRT-PCR.

### In vivo S1m precipitation

For in vivo pulldown of S1m-tagged DANCR, S1m vector or S1m-DANCR was transfected into BXPC-3 cells in a 10 cm dish. The cells were harvested 48 h after transfection and then lysed in 1 ml lysis buffer containing protein inhibitors and RNase inhibitor. The supernatant was collected after centrifugation for 20 min at 4 °C and then incubated with streptavidin beads (MilliporeSigma) for 30 min at 4 °C to remove the background. The pre-cleaned cell lysate was transferred to a new tube and 1/10 of the lysate was saved as protein input. The remaining lysate was incubated with streptavidin beads for 4 h at 4 °C before washing 5 times with ice-cold PBS. Finally, the pellet was dissolved in 30 µl 2xSDS sample buffer, followed by SDS-PAGE and Western blot with IGF2BP2 antibody.

To detect m6A modified DANCR from S1m pulldown, S1m-DANCR was pulled down as above, and then washed three times with RIP buffer. Next, the pellet was resuspended in 1 ml pulldown buffer and incubated with m6A antibody (2 µl) and incubated overnight at 4 °C with rotation. After five times of washes with ice cold PBS, the pellet was resuspended in 30 µl 2× SDS sample buffer. Finally, m6A antibody bound to S1m-DANCR was subject to SDS-PAGE and western blot; the signal was detected directly with donkey antimouse antibody (H + L) conjugated with IRDye 680.

### Animal experiment

BALB/c nude mice (4–6 weeks old) were purchased from Model Animal Research Center of Nanjing University (Nanjing, China), with the animal protocol (No. 2017KT081) approved by the animal care and ethics committee at Zhejiang Provincial People’s Hospital. BXPC-3 cells with DANCR overexpression or empty vector control; DANCR KO or empty vector control were harvested and then mixed with matrigel (1:1) (BD Biosciences). Three different numbers of cells (1 × 10^4^, 1 × 10^5^, and 5 × 10^5^ cells) were subcutaneously injected into nude mice, five animals per group. Tumor growth rate was measured every five days and the tumor volume was calculated as follows: tumor volume (mm^3^) = (length × width^2^)/2. The mice were sacrificed 3 weeks after injection.

For orthotopic pancreatic tumor mouse model, BXPC-3 cells carrying DANCR KO or vector control were harvested and then resuspended in RPMI 1640 medium. Nude mice were first anesthetized with sodium pentobarbital. BXPC-3 cells (1 × 10^6^ cells in 50 µl) were injected into the pancreases of nude mice by an insulin syringe (30 G), seven animals per group. The peritoneum and skin were closed with 4.0 surgical sutures. The mice were sacrificed 3 weeks after injection.

### Statistical analysis

All statistical analyses were performed using the GraphPad Prism program. The continuous variables are summarized as mean and standard error of mean (SEM) unless stated. The two-sample *t* test was used to compare the mean of a continuous variable between two samples. The multiple comparisons after repeated measures ANOVA was used to compare the entire curve of tumor growth. Association between two categorical variables was evaluated by using the Fisher’s exact test. OS and disease-free survival (DFS) curves were calculated with the Kaplan–Meier method and were analyzed with the log-rank test. All *P* values were two-sided and *P* values < 0.05 were considered as significant.

## Supplementary information

Supplementary figures

SF legend
