# Supplementary material for: IGF2BP2 regulates DANCR by serving as an N6-methyladenosine reader
Source: Cell Death Differ. 2019 Dec 5;27(6):1782–94. doi: 10.1038/s41418-019-0461-z (PMC7244758; doi:10.1038/s41418-019-0461-z)
Supplement: Supplementary file 1 — Supplementary figures [file 41418_2019_461_MOESM1_ESM.pdf]

Figure S1

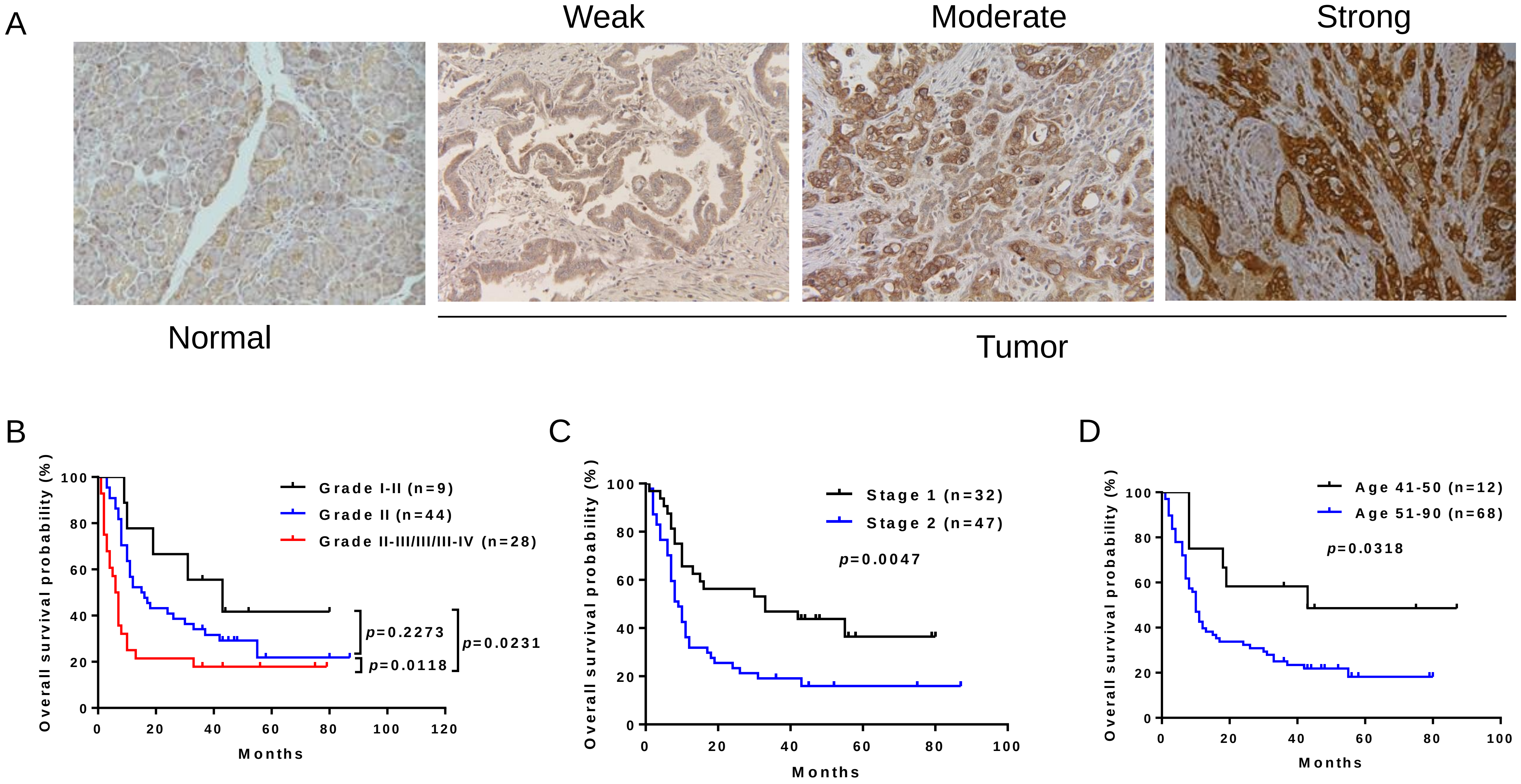
A
Weak
Moderate
Strong
Normal
Tumor
B
Overall survival probability (%)
Months
Grade I-II (n=9)
Grade II (n=44)
Grade II-III/III/III-IV (n=28)
p=0.2273
p=0.0118
p=0.0231
C
Overall survival probability (%)
Months
Stage 1 (n=32)
Stage 2 (n=47)
p=0.0047
D
Overall survival probability (%)
Months
Age 41-50 (n=12)
Age 51-90 (n=68)
p=0.0318

Figure S2

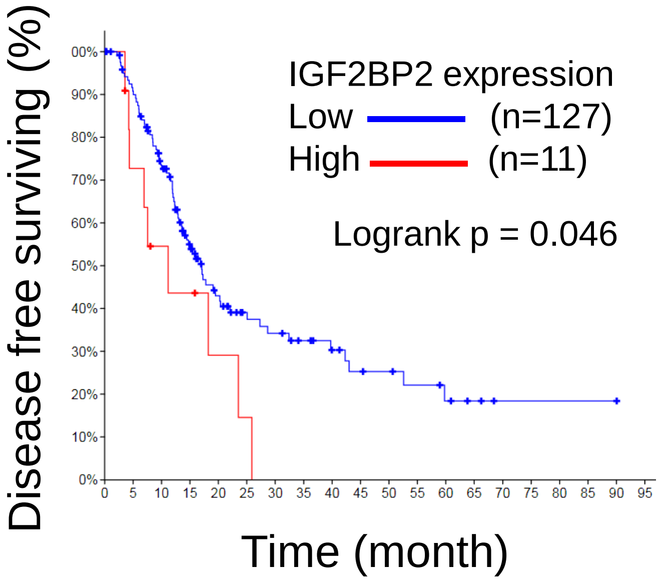

	Total cases	Relapsed/ Progressed	Median Months Disease-free
Cases with IGF2BP2 high	11	9	11.17
Cases with IGF2BP2 low	127	72	17.05

Figure S3

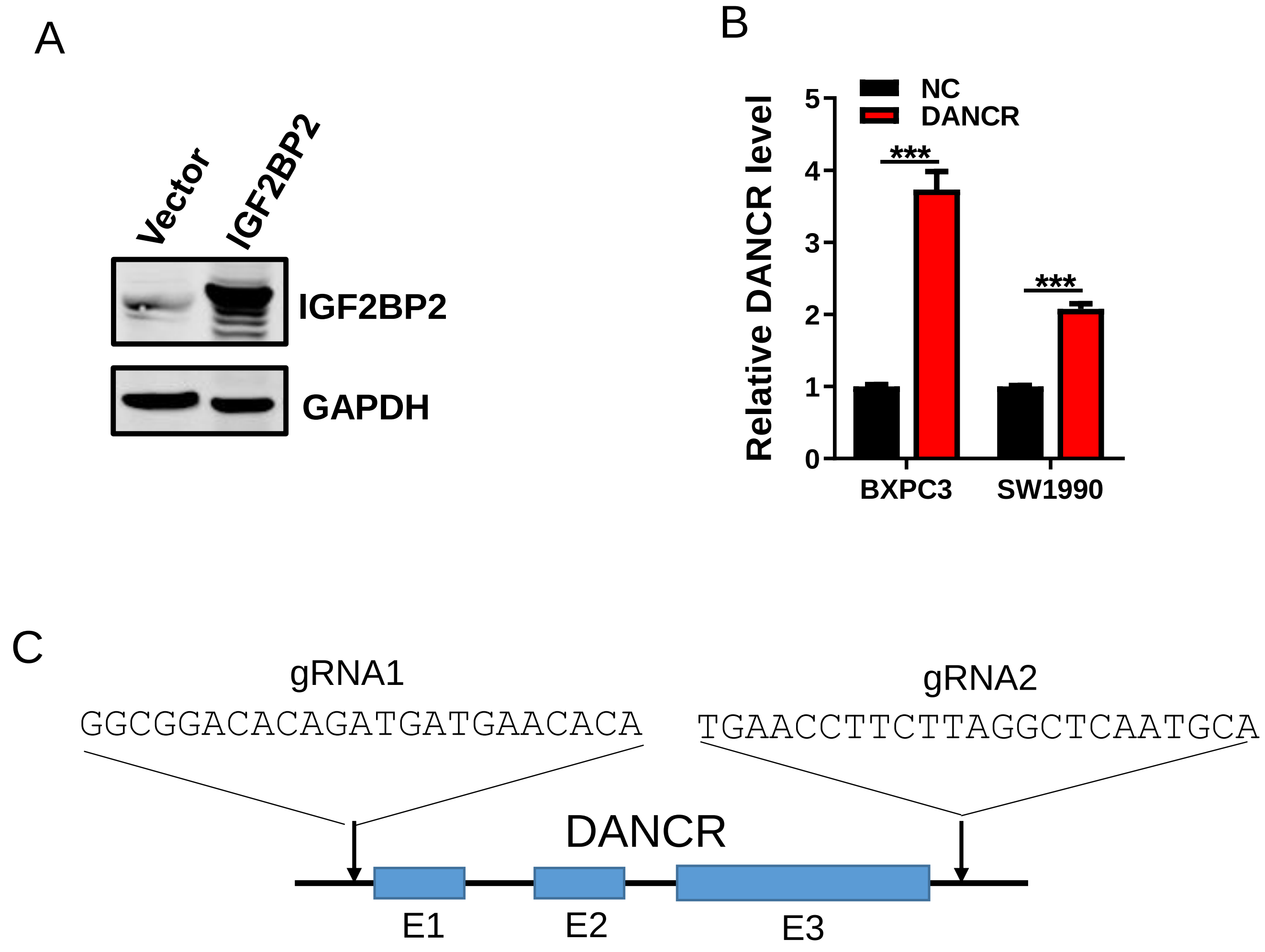

A
Vector
IGF2BP2
IGF2BP2
GAPDH
B
Relative DANCR level
NC
DANCR
***
***
0
1
2
3
4
5
BXPC3
SW1990
C
gRNA1
GGCGGACACAGATGATGAACACA
gRNA2
TGAACCTTCTTAGGCTCAATGCA
DANCR
E1
E2
E3

Figure S4

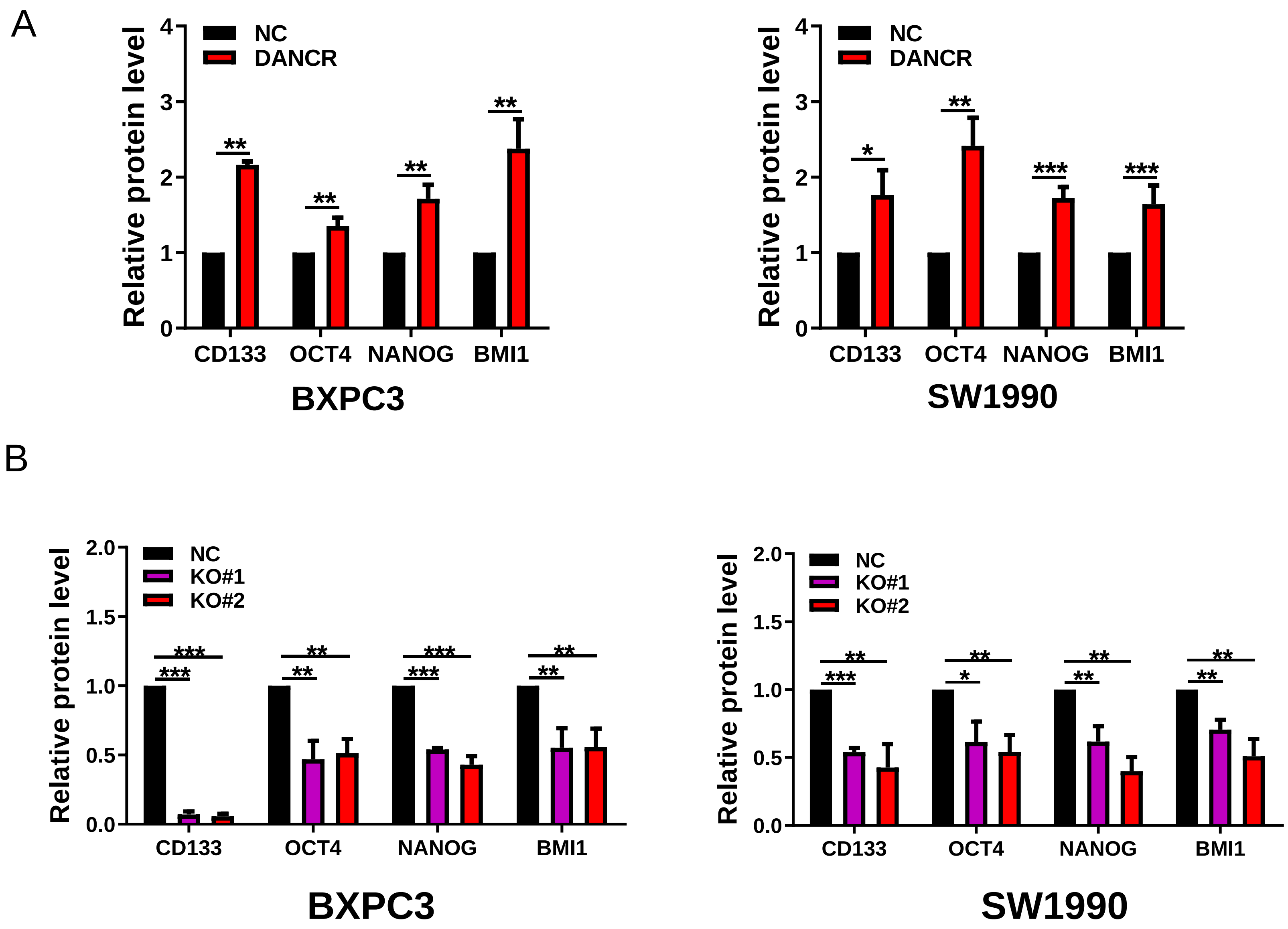
A
Relative protein level
NC
DANCR
CD133
OCT4
NANOG
BMI1
BXPC3
SW1990
B
NC
KO#1
KO#2
BXPC3
SW1990

Figure S5

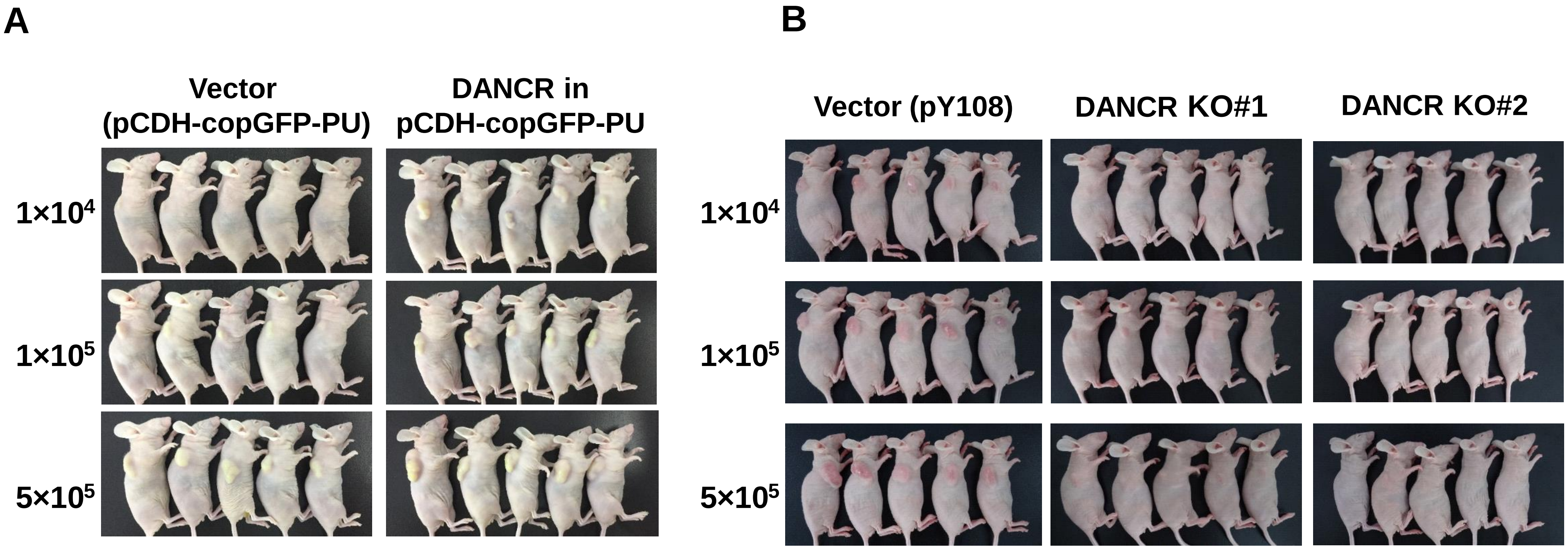

A
Vector (pCDH-copGFP-PU)
DANCR in pCDH-copGFP-PU
1×10^4
1×10^5
5×10^5
B
Vector (pY108)
DANCR KO#1
DANCR KO#2
1×10^4
1×10^5
5×10^5

Figure S6

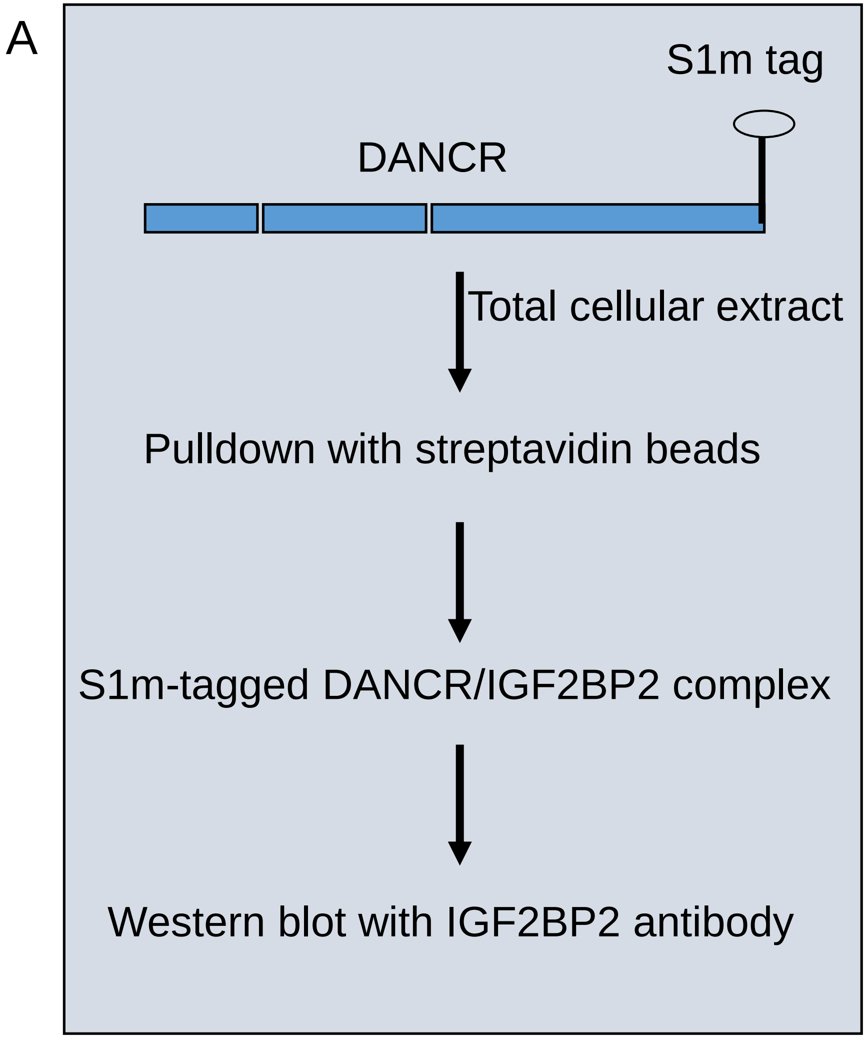
A
S1m tag
DANCR
Total cellular extract
Pulldown with streptavidin beads
S1m-tagged DANCR/IGF2BP2 complex
Western blot with IGF2BP2 antibody

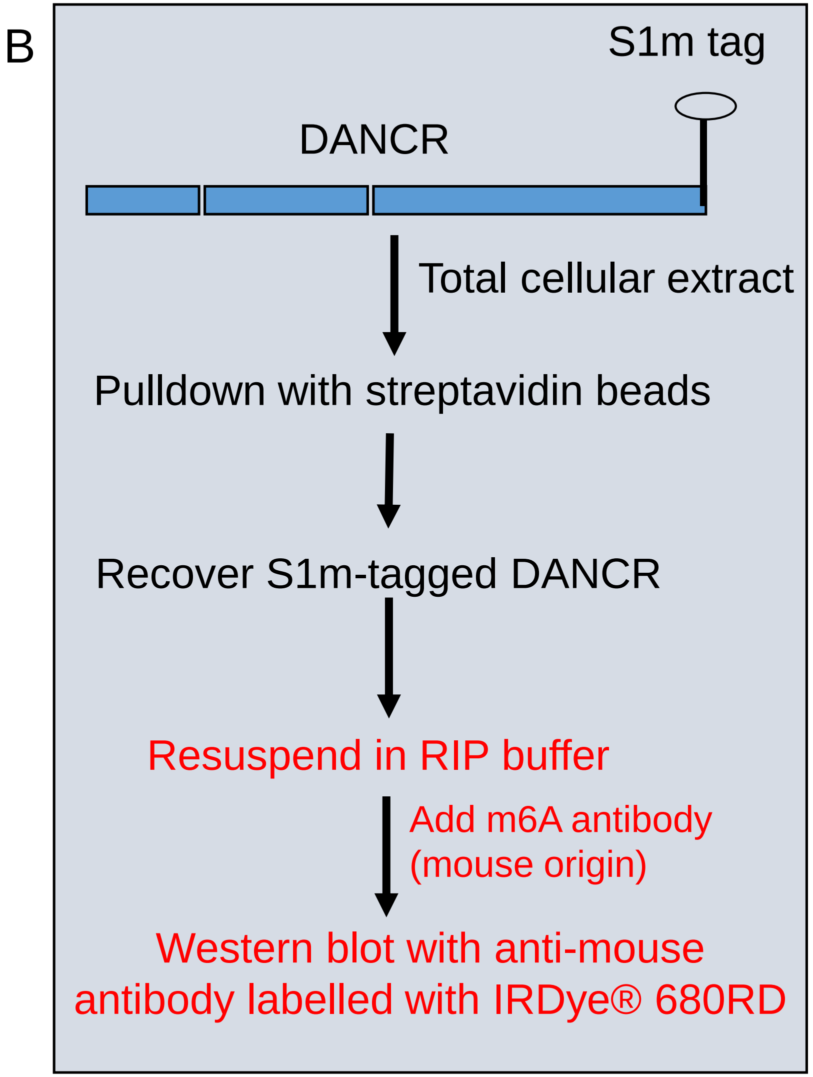
B
S1m tag
DANCR
Total cellular extract
Pulldown with streptavidin beads
Recover S1m-tagged DANCR
Resuspend in RIP buffer
Add m6A antibody
(mouse origin)
Western blot with anti-mouse
antibody labelled with IRDye® 680RD

Figure S7

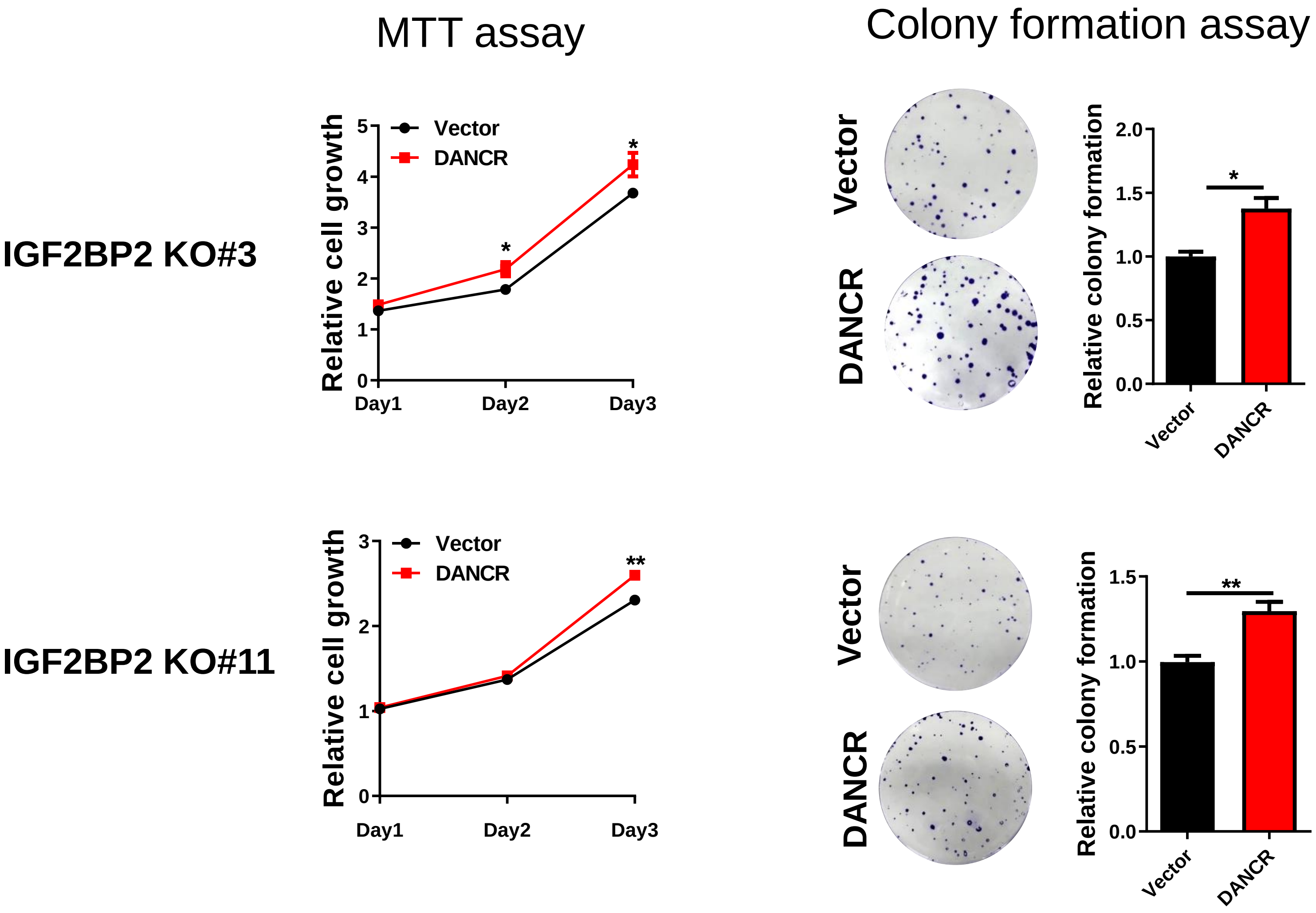

MTT assay
Colony formation assay
IGF2BP2 KO#3
IGF2BP2 KO#11
Relative cell growth
Vector
DANCR
Day1
Day2
Day3
0
1
2
3
4
5
*
*
**
Relative colony formation
0.0
0.5
1.0
1.5
2.0
Vector
DANCR
