# Supplementary material for: IGF2BP2 regulates DANCR by serving as an N6-methyladenosine reader
Source: Cell Death Differ. 2019 Dec 5;27(6):1782–94. doi: 10.1038/s41418-019-0461-z (PMC7244758; doi:10.1038/s41418-019-0461-z)
Supplement: Supplementary file 2 — SF legend [file 41418_2019_461_MOESM2_ESM.docx]

**Supplementary figure legend**

Figure S1. (A) Representative IHC pictures for different levels of IGF2BP2 expression. Clinical outcomes are associated with disease grade (A), stage (B) and age (C) based on Supplementary T the TMA slide.

Figure S2 High expression of IGF2BP2 is associated with poor disease free survival in pancreatic cancer based on Pancreatic Adenocarcinoma dataset (TCGA, Provisional) analysis of mRNA levels (high and low).

Figure S3. (A) Ectopic expression of IGF2BP2 in BXPC-3 cells as detected by western blot. (B) Detection of ectopically expressed IGF2BP2 by qRT-PCR in BXPC-3 and SW1990 cells. (C) Strategy for DANCR knockout by CRISPR/Cpf1 dual gRNAs.

Figure S4. Quantitative analysis of western blot for Figure 4H (A) and Figure 5E (B).

Figure S5. DANCR overexpression promotes whereas DANCR KO suppresses tumor progression. (A), Tumor bearing mice from Figure 6A before tumors were harvested. (B), Tumor bearing mice from Figure 6B before tumors were harvested.

Figure S6. (A) Detection of in vivo DANCR-IGF2BP2 interaction; (B) Detection of S1m-DANCR-m6A antibody interaction.

Figure S7. Effect of ectopic expression of DANCR on cell proliferation and viability in IGFBP2 KO BXPC-3 cells (KO#3 and #11), as determined by MTT and colony formation assays. Values are SEM. *, P < 0.05; **, P < 0.01.
